# Alleviation of allergic conjunctivitis by (±)5(6)-dihydroxy-8Z,11Z,14Z,17Z-eicosatetraenoic acid in mice

**DOI:** 10.3389/fphar.2023.1217397

**Published:** 2023-09-26

**Authors:** Nanae Nagata, Tomoka Suzuki, Shinya Takenouchi, Koji Kobayashi, Takahisa Murata

**Affiliations:** ^1^ Animal Radiology, Graduate School of Agricultural and Life Sciences, The University of Tokyo, Tokyo, Japan; ^2^ Food and Animal Systemics, Graduate School of Agricultural and Life Sciences, The University of Tokyo, Tokyo, Japan; ^3^ Veterinary Pharmacology, Graduate School of Agricultural and Life Sciences, The University of Tokyo, Tokyo, Japan

**Keywords:** (±)5(6)-DiHETE, allergic conjunctivitis, pollen, lipid, histamine

## Abstract

**Background:** Allergic conjunctivitis (AC) is a common ophthalmologic disorder that causes symptoms that often reduces a patient’s quality of life (QOL). We investigated the effects of the eicosapentaenoic acid metabolite (±)5(6)-dihydroxy-8Z,11Z,14Z,17Z-eicosatetraenoic acid ((±)5(6)-DiHETE) on AC using a mouse model.

**Methods:** BALB/c mice were sensitized with two injections of short ragweed pollen in alum, challenged fifth with pollen in eyedrops. The clinical signs and tear volume were evaluated at 15 min after the final challenge. Histamine-induced ocular inflammation model was prepared by instilling histamine onto the surface of the eye. Fifteen minutes after histamine application, tear volume was measured using the Schirmer tear test. Miles assay was performed to investigate vascular permeability. To cause scratching behavior 10 μg of serotonin was injected in the cheek.

**Results:** Repeated topical application of pollen induced conjunctivitis, accompanied by eyelid edema and tearing in mice. Pollen application typically degranulates mast cells and recruits eosinophils to the conjunctiva. Intraperitoneal administration of 300 μg/kg of (±)5(6)-DiHETE significantly inhibited pollen-induced symptoms. The administration of (±)5(6)-DiHETE also attenuated mast cell degranulation and eosinophil infiltration into the conjunctiva. To assess the effects of (±)5(6)-DiHETE on the downstream pathway of mast cell activation in AC, we used a histamine-induced ocular inflammation model. Topical application of 4 μg/eye histamine caused eyelid edema and tearing and increased vascular permeability, as indicated by Evans blue dye extravasation. Intraperitoneal administration of 300 μg/kg or topical administration of 1 μg/eye (±)5(6)-DiHETE inhibited histamine-induced manifestations. Finally, we assessed the effects of (±)5(6)-DiHETE on itching. An intradermal injection of 10 μg serotonin in the cheek caused scratching behavior in mice. Intraperitoneal administration of 300 μg/kg (±)5(6)-DiHETE significantly inhibited serotonin-induced scratching.

**Conclusion:** Thus, (±)5(6)-DiHETE treatment broadly suppressed AC pathology and could be a novel treatment option for AC.

## 1 Introduction

Allergic conjunctivitis (AC), a common ophthalmologic disorder affecting up to 40% of the general population (Singh K et al., 2010; Miyazaki et al., 2020), causes several symptoms, such as conjunctival hyperemia, lacrimation and ocular itching. During the period of antigen exposure, these symptoms significantly reduce the patient’s quality of life (QOL). There are two main types of AC: seasonal and perennial. During seasonal AC, symptoms are observed at a fixed time every year. Most seasonal cases of AC are caused by pollen. Perennial AC is not seasonal and tends to be chronic. Mites and house dust are the major causative antigens of perennial AC.

AC includes two phases of the inflammatory response. In the early phase of AC, an immunoglobulin E (IgE)-dependent inflammatory response occurs within approximately 15 min after allergen exposure. Mast cells activated by the allergen-IgE complex release inflammatory mediators such as histamine and serotonin. These mediators can cause conjunctival hyperemia, lacrimation, eyelid swelling and ocular itching. The late phase of AC usually occurs 12–24 h after allergen exposure. In this phase, eosinophils and T-helper 2 cells infiltrate the conjunctiva and release cytokines, including IL-4, IL-5 and IL-13, which exacerbate and prolong the symptoms of AC (Magone MT et al., 1998; Hirakata et al., 2019a).

First-line AC treatment consists of topical anti-histamines and mast cell stabilizers. However, these treatments sometimes fail to provide satisfactory relief, particularly in the chronic phase. Although anti-inflammatory steroids are another option for the treatment of AC (Sacta et al., 2016), they have side effects, including increased intraocular pressure and infection (Miyazaki et al., 2020). Therefore, a more effective and safer treatment with a novel mechanism of action is required, especially for the control of chronic and severe AC.

Lipid mediators are small molecules derived mainly from ω-6 or ω-3 polyunsaturated fatty acids (PUFAs) via enzymatic or non-enzymatic oxidation processes. The lipid mediators produced regulate the progression and resolution of inflammation. The intake or treatment of ω-6 PUFA and its derivatives, lipid mediators such as prostaglandins and leukotrienes often represent pro-inflammatory or anti-inflammatory reactions in various types of diseases. Recent studies have focused on the anti-inflammatory effects of ω-3 PUFAs including docosahexaenoic acid (DHA), eicosapentaenoic acid (EPA) and their derivatives. Indeed, several studies have reported that dietary ω-3 fatty acids alleviate AC in mouse models (Hirakata et al., 2019b; Hamazaki et al., 2019). Hirakata et al. (2019a) showed that a ω-3 fatty acid diet reduced the levels of various inflammatory lipid mediators in the conjunctival tissue of AC mice. The DHA derivative resolvin D1 attenuates murine AC induced by an excretory-secretory protein of *Acanthamoeba* (Kang MS et al., 2021). However, the mechanism underlying the anti-inflammatory effects of ω-3 PUFAs and their derivatives remains unclear.

CYP450 monooxygenases catalyzes the epoxygenation of EPA. Epoxygenase products are then metabolized via soluble epoxide hydrolase (sEH) to form dihydroxy FAs such as DiHETE (Konkel and Schunck, 2011). Previously, our group found that the level of (±)5(6)-DiHETE increased in the colon of dextran sulfate sodium (DSS)-induced colitis mice and that post-administration of (±)5(6)-DiHETE promoted recovery from colitis (Hamabata et al., 2018a; Hamabata et al., 2018b; Kobayashi et al., 2021a). We also found that treatment with (±)5(6)-DiHETE attenuated histamine-induced vascular hyperpermeability, at least partially, by inhibiting the activation of transient receptor potential vanilloid (TRPV) 4 channels (Hamabata et al., 2018a; Kobayashi et al., 2021a). TRPV4 is a non-selective cation channel responsible for sensing osmotic and mechanical signals and regulating calcium signaling across cell membranes. Accumulating evidence shows that TRPV4 activation is involved in various types of cells, including mast cells, vascular endothelial cells and nerve cells. These observations allowed us to hypothesize that the EPA derivative (±)5(6)-DiHETE may attenuate the symptoms of AC. In this study, we aimed to investigate the therapeutic potential of (±)5(6)-DiHETE in pollen- or histamine-induced AC in mice.

## 2 Materials and methods

### 2.1 Animals

Male BALB/c mice were purchased from CLEA Japan (Tokyo, Japan). Mice were maintained in isolated cages under a 12-h light/12-h dark photoperiod. Water and food were provided *ad libitum*. All experiments were approved by the Institutional Animal Care and Use Committee of the University of Tokyo.

### 2.2 Pollen-induced conjunctivitis model

Allergic conjunctivitis model was generated as described previously (Stern et al., 2005; Tanaka et al., 2015). Briefly, pollen from short ragweed (Polysciences, PA, United States) was emulsified with Imject Alum Adjuvant (Thermo Fisher Scientific, Tokyo, Japan). 6–8 weeks old wild-type male BALB/c mice were sensitized on day 0 by applying 50 μL of pollen (50 μg pollen with 50 μL alum) into the left hind foot. Five days later (day 5), a second sensitization was performed by injecting the reagents into the left hind foot. On days 10–14, the eyes of the mice were topically challenged daily with pollen in saline (2 mg per 10 μL/eye). On day 14, 15 min after the final eye drop challenge, the tear volume was measured using the Schirmer tear test. Fifteen minutes after the Schirmer test, the eyeballs (with lids and conjunctival tissue) were collected.

(±)5(6)-DiHETE (Cayman, MI, United States) (300 μg/kg) or dexamethasone sodium phosphate (DEX) (FUJIFILM Wako, Tokyo, Japan) (2 mg/kg) was intraperitoneally injected into mice on days 10–14. We determined the doses of (±)5(6)-DiHETE based on our previous research (Kobayashi et al., 2021a; Takenouchi et al., 2021). The severity of AC symptoms was evaluated using the total points scored for conjunctival redness, chemosis, lid edema and tearing as follows: 0, none; 1, mild; and 2, severe.

### 2.3 May-Grunwald Giemsa staining

Dissected eyeballs (with lids and conjunctival tissue) were fixed in 4% paraformaldehyde (PFA) for 24 h and embedded in paraffin. Tissue sections (4 μm) were prepared and stained with May-Grunwald Giemsa. Briefly, the sections were immersed in May-Grunwald stain solution (FUJIFILM Wako, 1:20 in 1/150 M phosphate-buffer, pH6.7) for 5 min and then in diluted Giemsa solution (FUJIFILM Wako, 1:20 in H_2_O) for 5 min. May-Grunwald Giemsa-positive mast cells and eosinophils were counted in five randomly selected fields (×400 magnification) per section.

### 2.4 Histamine-induced conjunctivitis model

Histamine-induced conjunctivitis was induced as described previously, with some modifications (Mochizuki et al., 2022). Briefly, histamine (4 μg in 4 μL/eye, FUJIFILM Wako) was dropped onto the surface of the eye. Fifteen minutes after histamine application, tear volume was measured using the Schirmer tear test. To investigate vascular permeability, Evans blue (SIGMA, Tokyo, Japan, 10 mg/mL, 0.1 mL) was injected intravenously 5 min after histamine challenge. The eyeballs (with lids and conjunctival tissue) were harvested 15 min after the histamine challenge, dried at 55°C and weighed.

In some experiments, (±)5(6)-DiHETE (300 μg/kg) or ketotifen (SIGMA, 10 mg/kg) was intraperitoneally injected into mice 15- or 30-min prior to histamine (4 μg/eye) administration, respectively. In other experiments, (±)5(6)-DiHETE (1 μg in 2 μL/eye) or ketotifen (0.1 μg in 2 μL/eye) was dropped onto the eye 15- and 45-min prior to histamine administration (8 μg in 4 μL/eye).

### 2.5 Measurement of Evans blue concentration in the conjunctiva by Miles assay

The extravasated Evans blue present in the harvested eyeball was extracted for 18 h by placing the dissected eye tissues in 1 mL of 1 N KOH at 40°C. After neutralization, the dye was extracted with acetone (7 mL). After drying *in vacuo*, the resulting residue was reconstituted in 25 μL DMSO. The amount of dye was estimated by measuring the absorbance at 620 nm.

### 2.6 Serotonin-induced scratching

The mice were acclimatized to the recording room for at least 1 h before the experiments. The scratching behavior was observed as described previously (Shimada and LaMotte, 2008; Kobayashi et al., 2021b). Briefly, 10 μg serotonin in 10 μL saline was injected intradermally (i.d.) into the mouse cheek. Mouse behavior was recorded for 30 min to assess scratching. One bout of scratching was defined as an episode in which a mouse lifted its hind paw and scratched continuously for any length of time until the paw returned to the floor or mouth for licking. We evaluated scratching behavior by counting the number of paw episodes brought to the cheek. HC-067047 (HC) was dissolved in 10% DMSO and 10% tween20 in saline. (±)5(6)-DiHETE (300 μg/kg) or HC (20 mg/kg) was intraperitoneally injected into mice immediately before serotonin injection.

### 2.7 Statistics

Statistical analyses were performed using Bell Curve for Excel software (Social Survey Research Information Co., Ltd. Tokyo, Japan). Statistical differences between the control and test groups were analyzed using Student’s t-test for two-group comparisons and one-way ANOVA with Tukey’s test for multiple-group comparisons. Data are expressed as the mean ± SEM. Statistical significance was defined as *p < 0.05, **p < 0.01.

## 3 Results

### 3.1 Attenuation of allergic symptoms by (±)5(6)-DiHETE

**FIGURE 1:**
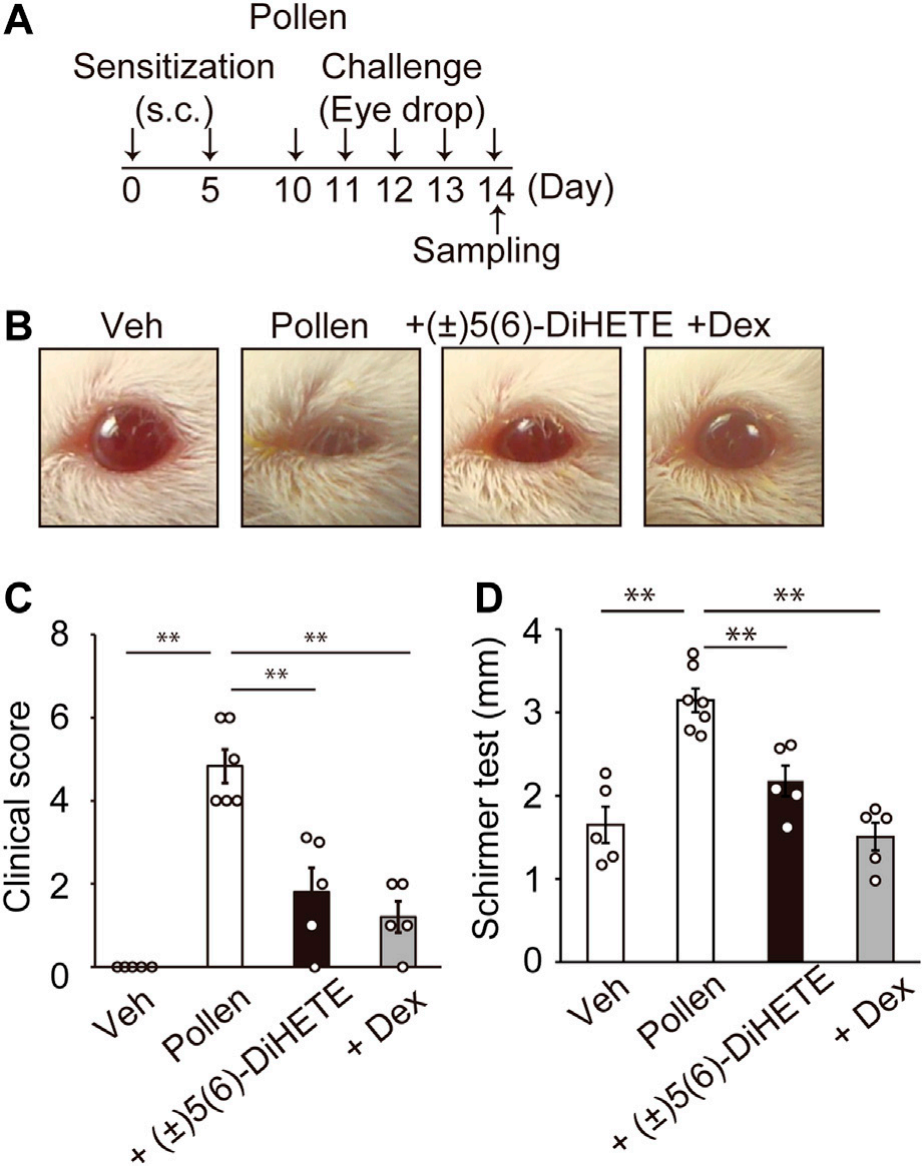
Improvement of allergic symptoms by (±)5(6)-DiHETE **(A)** Protocol for pollen-induced AC. **(B)** Representative pictures of mouse eyes on day 14, at 15 min after the final pollen application. **(C)** The clinical scores at 20 min after the final pollen application. The total score consisted of the sum of scores for 3 factors (eyelid edema, redness and tearing) each scored from 0 to 3 points, depending on the severity (*n* = 5–6). Intraperitoneal administration of (±)5(6)-DiHETE (300 μg/kg) or dexamethasone (DEX, 2 mg/kg) from day 10 to day 14 reduced clinical scores. **(D)** Schirmer’s test was performed to evaluate the tear volume at 15 min after the final pollen application (*n* = 5–7). Intraperitoneal administration of (±)5(6)-DiHETE (300 μg/kg) or DEX (2 mg/kg) from day 10 to day 14 reduced tear volume. Data are presented as a mean ± SEM. **p < 0.01.

In the AC mouse model, early phase allergic symptoms occurred approximately 20 min after the antigen challenge (Magone MT et al., 1998). We first generated a murine model of AC induced by the repeated topical application of ragweed pollen to the eye. Mice were sensitized twice on days 0 and 5 by subcutaneously injecting pollen, then challenged five more times with eye drops containing pollen once daily from days 10–14 (Figure 1A). Figure 1B shows typical images of mouse eyes 20 min after the 5th pollen application. Pollen application induced conjunctivitis accompanied by eyelid edema, conjunctiva redness and tearing. Some mice (3 in 6) closed their eyes after pollen application, likely because of pain and itching. To evaluate the severity of AC symptoms, we compared clinical scores based on eyelid edema, redness, and tearing was scored (Figure 1C). The application of pollen significantly increased the score on day 14 (vehicle, 0.0 ± 0.0; pollen, 4.8 ± 0.4). Intraperitoneal administration of 300 μg/kg (±)5(6)-DiHETE or 2 mg/kg of DEX from day 10 to day 14 decreased increased clinical scores by 62.8% or 75.2%, respectively [(±)5(6)-DiHETE, 1.8 ± 0.6; DEX, 1.2 ± 0.4]. These treatments ameliorated pollen-induced eye closing. Schirmer’s test was performed to evaluate the tear volume on day 14, at 15 min after the final pollen eye drop applications. We also confirmed that the administration of (±)5(6)-DiHETE did not change the appearance or behavior of mice 30 min after administration. The pollen application increased tearing by 190% compared with the vehicle application (vehicle, 1.65 ± 0.22 mm; pollen, 3.14 ± 0.14 mm) and intraperitoneal administration of (±)5(6)-DiHETE or DEX attenuated this increment by 64.8% or 110%, respectively [(±)5(6)-DiHETE, 2.18 ± 0.19 mm or DEX, 1.51 ± 0.17 mm] (Figure 1D). No significant differences between (±)5(6)-DiHETE and DEX were observed in clinical score and tearing. These results suggest that (±)5(6)-DiHETE suppressed a variety of allergic symptoms that occur in the early phases of AC.

### 3.2 Attenuation of mast cell degranulation by (±)5(6)-DiHETE in the conjunctiva

**FIGURE 2:**
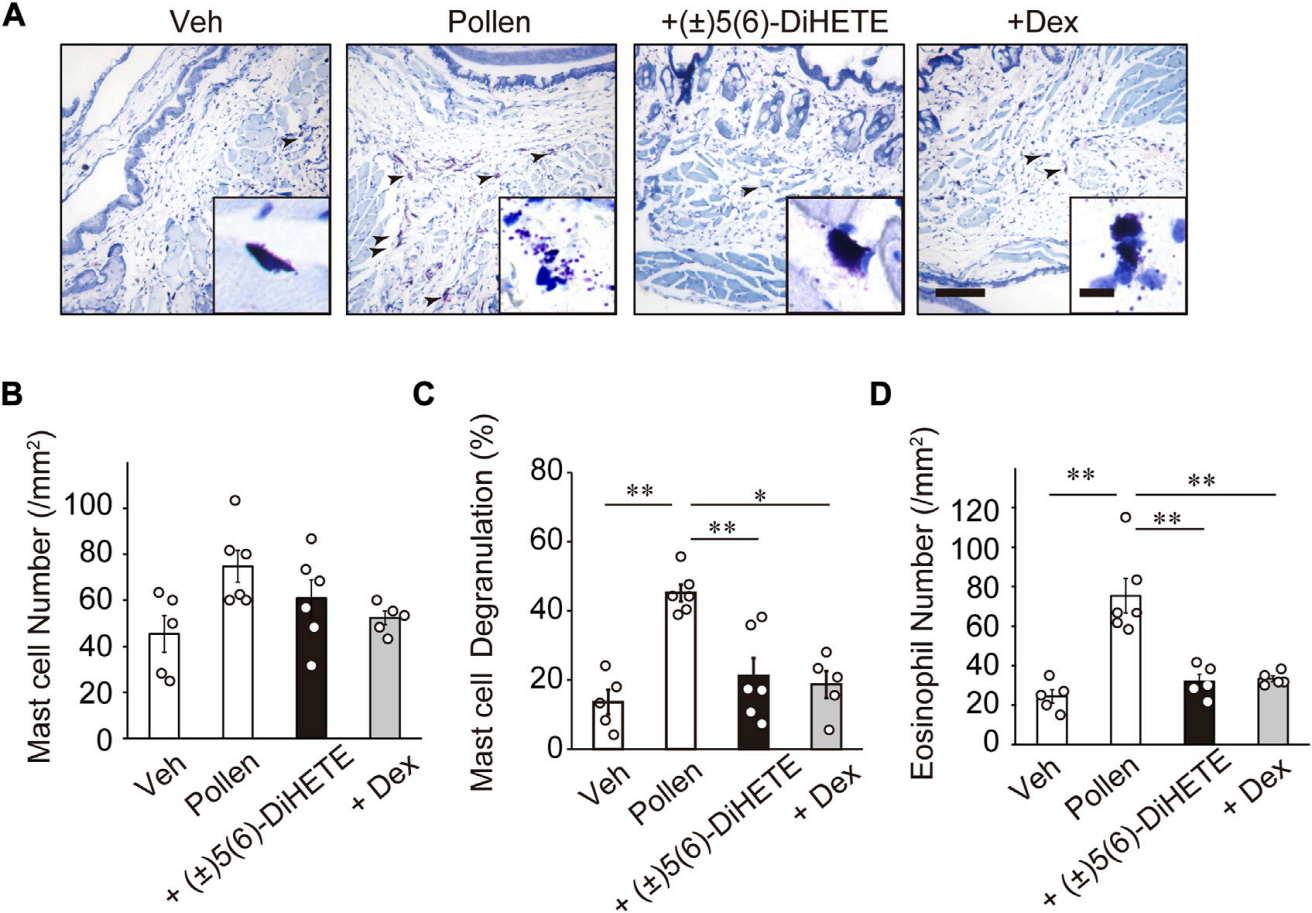
Attenuation of degranulation of mast cells and infiltration of eosinophils into the conjunctiva by (±)5(6)-DiHETE **(A)** Representative pictures of May-Grunwald Giemsa staining of the conjunctiva on day 14 and 30 min after the final pollen application. Arrowheads indicate degranulated mast cells (scale bar, 100 μm; inset 10 μm). **(B)** Pollen application did not alter the number of mast cells in the conjunctiva. Intraperitoneal administration of (±)5(6)-DiHETE (300 μg/kg) or dexamethasone (DEX, 2 mg/kg) from days 10–14 reduced the number of degranulated mast cells [**(C)**, *n* = 5–6] and infiltrated eosinophils [**(D)**, *n* = 5–6] in the conjunctiva. Data are presented as a mean ± SEM. *p < 0.05. **p < 0.01.

To investigate the mechanism by which (±)5(6)-DiHETE alleviates allergic symptoms, we evaluated mast cell degranulation in the conjunctiva. Figure 2A shows typical images of May-Grunwald Giemsa staining.

Mast cells appeared in deep purple color with many granules filled in the cytoplasm and the shape of the cells varied from spindle-shape to circle. When mast cells had degranulated, the granules were observed near the cells. Pollen application did not alter the number of mast cells in the conjunctiva (Figure 2B). The rate of mast cell degranulation increased in the conjunctiva of pollen-treated mice by 332% (vehicle, 13.6% ± 3.5%; pollen, 45.2% ± 2.5%) (Figure 2C). Intraperitoneal administration of (±)5(6)-DiHETE or DEX significantly reduced this increase by 76.3% and 83.9%, respectively [(±)5(6)-DiHETE, 21.1% ± 5.3% or DEX, 18.7% ± 3.9%]. No significant differences between (±)5(6)-DiHETE and DEX were observed in the number of degranulated mast cells. These results suggest that (±)5(6)-DiHETE attenuated pollen-induced degranulation of mast cells in the conjunctiva, resulting in the relief of AC symptoms.

### 3.3 Attenuation of the infiltration of eosinophils into the conjunctiva by (±)5(6)-DiHETE

In the early phase of onset of AC, mast cell degranulation releases inflammatory mediators, leading to the infiltration of eosinophils into the conjunctiva. Infiltrated eosinophils promote late-phase allergic symptoms by releasing pro-inflammatory mediators (Stone et al., 2010). The pollen applications increased the number of eosinophils in the conjunctiva by 309% on day 14 at 30 min after the 5th pollen application (vehicle, 24.4 ± 3.4 cells/mm^2^; pollen, 75.3 ± 8.7 cells/mm^2^) (Figure 2D). Intraperitoneal administration of (±)5(6)-DiHETE or DEX reduced the number of infiltrated eosinophils by 85.0% or 82.3%, respectively [(±)5(6)-DiHETE, 32.0 ± 3.6 cells/mm^2^ or DEX, 33.4 ± 1.5 cells/mm^2^]. These results suggest that (±)5(6)-DiHETE inhibited mast cell degranulation, resulting in the reduction of eosinophil infiltration into the conjunctiva and suppression of late-phase symptoms of AC. These results suggest that the reduction in eosinophil infiltration was at least partially due to the inhibition of mast cell degranulation by (±)5(6)-DiHETE.

### 3.4 Attenuation of histamine-induced inflammation by (±)5(6)-DiHETE

**FIGURE 3:**
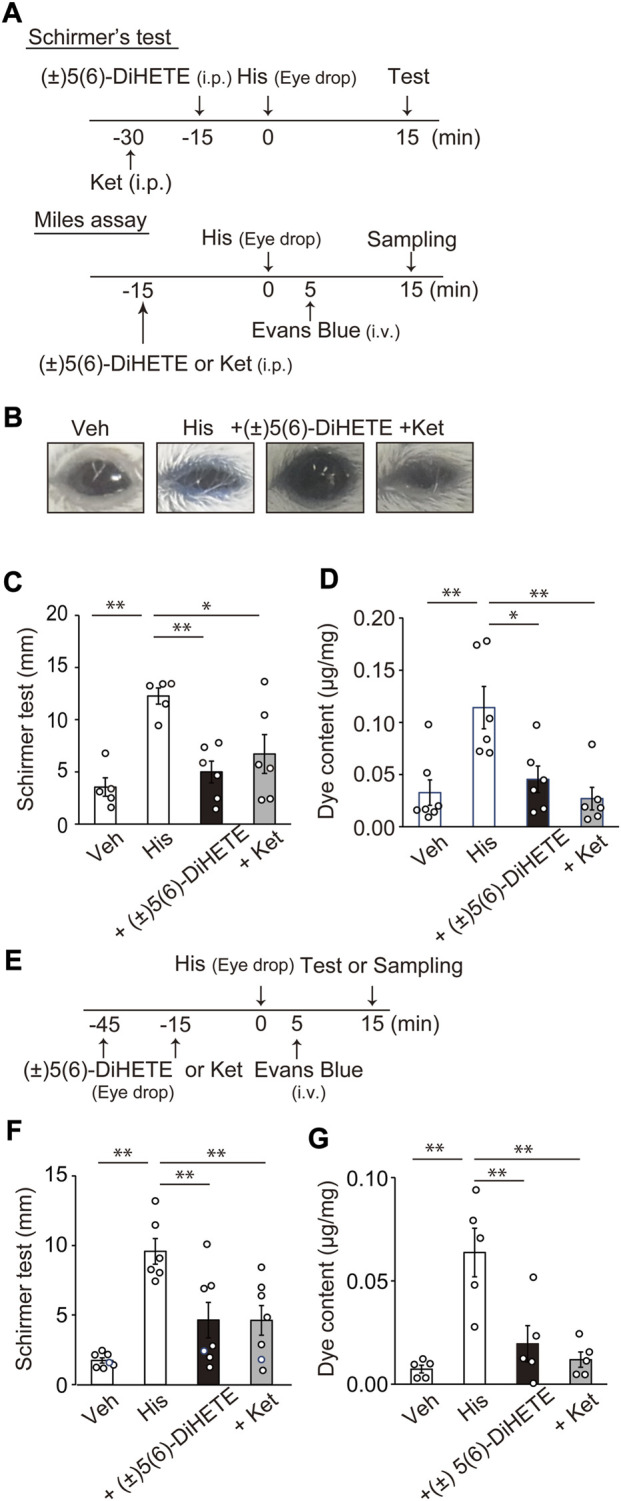
Improvement of histamine-induced ocular inflammation by (±)5(6)-DiHETE **(A)** Protocol for intraperitoneal administration of (±)5(6)-DiHETE in histamine-induced ocular inflammation. **(B)** Representative pictures of the mouse eyes 15 min after histamine application in the Miles assay. Tear volume was evaluated by Schirmer’s test. Intraperitoneal administration of (±)5(6)-DiHETE (300 μg/kg) or ketotifen (Ket, 10 mg/kg) reduced tear volume at 15 min after histamine application [**(C)**, *n* = 5–6] and the Evans blue dye extravasation in the eyeballs [**(D)**, *n* = 6–7] at 15 min after histamine application. **(E)** Protocol for topical administration of (±)5(6)-DiHETE in histamine-induced ocular inflammation. Topical administration of (±)5(6)-DiHETE (1 μg in 2 μL/eye) or ketotifen (0.1 μg in 2 μL/eye) reduced the tear volume [**(F)**, *n* = 6–7] and the Evans blue dye extravasation in the eyeballs [**(G)**, *n* = 5] at 15 min after histamine application. Data are presented as a mean ± SEM. *p < 0.05. **p < 0.01.

To confirm the effect of (±)5(6)-DiHETE on the downstream pathogenesis of mast cell activation (histamine release) in AC, we investigated the effect of (±)5(6)-DiHETE on histamine-induced conjunctivitis. Figure 3A shows a schematic representation of the experimental design. Topical application of 4 μg histamine to mouse eyes induced an inflammatory response characterized by conjunctival hyperemia, eyelid edema and tearing. We performed Schirmer’s test to assess tearing 15 min after applying histamine and the modified Miles assay to assess the effects of (±)5(6)-DiHETE on vascular permeability. Figure 3B shows typical images of the mouse eyes 15 min after histamine application in the Miles assay. Topical histamine application induced blue dye extravasation. Some mice (3 in 6) closed their eyes after histamine application, which may have been due to itching. Following histamine application, the mice opened their eyes after the application of (±)5(6)-DiHETE but not the histamine H1 receptor antagonist, ketotifen. Figure 3C shows the results of the Schirmer’s test. Topical histamine application increased tearing by 346% compared to the vehicle treatment (vehicle, 3.55 ± 0.88 mm; histamine, 12.27 ± 0.77 mm). Intraperitoneal administration of (±)5(6)-DiHETE (300 μg/kg, 15 min before) or ketotifen (10 mg/kg, 30 min before) attenuated the histamine-induced increase in tearing by 83.5% and 63.7%, respectively [(±)5(6)-DiHETE, 4.99 ± 1.05 or ketotifen, 6.71 ± 1.85 mm]. Figure 3D shows the results of quantification of dye extravasation. Topical histamine application also increased vascular permeability, as indicated by Evans blue dye extravasation (vehicle, 0.033 ± 0.012 μg/mg; His, 0.114 ± 0.020 μg/mg). Intraperitoneal administration of 300 μg/kg (±)5(6)-DiHETE (15 min before) reduced dye extravasation by 84.4% and 10 mg/kg ketotifen (30 min before) inhibited it by 107% [(±)5(6)-DiHETE, 0.045 ± 0.013 or ketotifen, 0.027 ± 0.011 μg/mg]. No significant differences were observed between (±)5(6)-DiHETE and ketotifen in terms of tearing and dye extravasation.

Considering its practical use, we investigated the effect of the topical administration of (±)5(6)-DiHETE. Figure 3E shows a schematic representation of the experimental design. Similar to the observations in intraperitoneal administration, topical administration of (±)5(6)-DiHETE (1 μg/eye, 15 min before) or ketotifen (0.1 μg/eye, 45 min before) decreased tearing by 63.0% or 63.2%, respectively [histamine, 9.58 ± 0.93; (±)5(6)-DiHETE, 4.63 ± 1.27 and ketotifen, 4.61 ± 1.06 mm] (Figure 3F). (±)5(6)-DiHETE and ketotifen also attenuated dye extravasation by 78.3% and 91.9%, respectively [histamine, 0.064 ± 0.012; (±)5(6)-DiHETE, 0.020 ± 0.009 and ketotifen, 0.012 ± 0.004 μg/mg] (Figure 3G). These results suggest that topical administration of (±)5(6)-DiHETE attenuated histamine-induced inflammatory responses.

### 3.5 Attenuation of serotonin-induced scratching behavior by (±)5(6)-DiHETE

**FIGURE 4:**
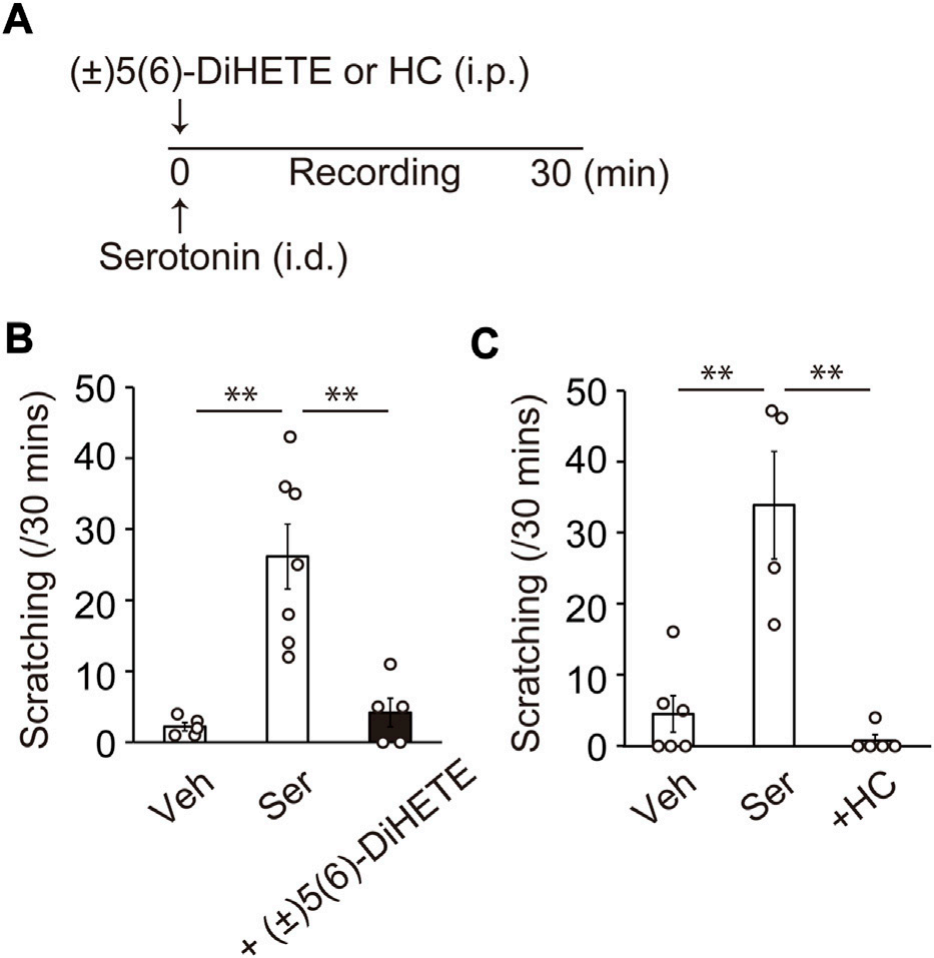
Improvement of allergic symptoms by (±)5(6)-DiHETE **(A)** Protocol for intraperitoneal administration of (±)5(6)-DiHETE in serotonin-induced scratching model. Intradermal injection of 10 μg serotonin (Ser) induced scratching behavior toward the cheek. Intraperitoneal administration of (±)5(6)-DiHETE (300 μg/kg) [**(B)**, *n* = 5–7] and TRPV4 antagonist HC-067047 (HC, 20 mg/kg) [**(C)**, *n* = 4–6] immediately before serotonin injection reduced serotonin-induced scratching behavior. Data are presented as a mean ± SEM. **p < 0.01.

Itch is a major and serious complaint in patients with AC. Scratching behavior was not observed in the current pollen-induced AC model. Since serotonin is known to be a much more potent inducer of scratching in mice than histamine (Kuraishi et al., 1995; Yamaguchi et al., 1999), we used a serotonin-induced scratching model. Figure 4A shows the schematic of the experimental design. Intradermal injection of 10 μg serotonin induced scratching behavior toward the cheek (vehicle, 2.2 ± 0.6; serotonin, 26.1 ± 4.6 counts/30 min) (Figure 4B). Intraperitoneal administration of 300 μg/kg (±)5(6)-DiHETE immediately before serotonin injection reduced scratching behavior by 91.6% (4.2 ± 2.0 count/30 min) (Figure 4B). Intraperitoneal administration of a TRPV4 antagonist HC-067047 HC, (20 mg/kg) inhibited the scratching behavior by 113% (0.8 ± 0.8 counts/30 min) (Figure 4C). These results suggest that (±)5(6)-DiHETE has the potential to suppress a variety of symptoms of AC.

## 4 Discussion

In this study, we demonstrated the potential of (±)5(6)-DiHETE as a novel treatment option for AC. In a pollen-induced AC mouse model, the intraperitoneal administration of 300 μg/kg (±)5(6)-DiHETE partially inhibited the degranulation of mast cells in the conjunctiva, strongly inhibited the infiltration of eosinophils into the conjunctiva and attenuated AC symptoms. In a histamine-induced ocular inflammation model, intraperitoneal (300 μg/kg) or topical (1 μg/eye) administration of (±)5(6)-DiHETE showed beneficial effects against tearing and increased vascular permeability, as indicated by Evans blue dye extravasation. Furthermore, 300 μg/kg (±)5(6)-DiHETE attenuated serotonin-induced scratching behavior in mice.

In this study, we used racemic mixture of 5(6)-DiHETE. Although we previously reported that 5(S),6(S)-DiHETE and 5(S),6(R)-DiHETE did not inhibit histamine-induced endothelial barrier dysfunction, further studies are required to clarify which enantiomer (or if both) is biologically active in AC models.

In the AC model, the treatment of (±)5(6)-DiHETE inhibited mast cell degranulation, which presumably lead to decrease of histamine release. We previously reported that administration of (±)5(6)-DiHETE inhibits histamine-induced vascular permeability enhancement (Hamabata et al., 2018b). Thus, (±)5(6)-DiHETE may attenuate both histamine release and histamine-mediated responses.

Activated mast cells produce large amounts of inflammatory mediators and promote eosinophil infiltration. The administration of (±)5(6)-DiHETE inhibited mast cell degranulation. The suppression of eosinophil infiltration observed at the same time is attributed, at least in part, to the suppression of mast cell degranulation by (±)5(6)-DiHETE. On the other hand, it is possible that (±)5(6)-DiHETE directly suppresses eosinophil activity and infiltration, and further studies are needed.

Besides (±)5(6)-DiHETE, there are several types of DiHETEs including (±)8,9-, (±)11,12-, and (±)14,15-DiHETEs (Knapp et al., 1991). Although we previously reported that (±)8,9-DiHETE did not inhibit histamine-induced endothelial barrier dysfunction, further studies are required to elucidate the pathophysiological roles of other types of DiHETEs.

Although anti-histamines or mast cell stabilizers are often used to treat AC, their effects are sometimes insufficient to control AC symptoms, especially in the chronic phase. In addition, allergic contact dermatitis has been reported as an adverse event caused by the anti-histamine drug ketotifen fumarate contained in eye drops (Romita et al., 2020). Steroid drugs are also used to treat severe AC symptoms, but they have side effects, including elevation of intraocular pressure and infection (Miyazaki et al., 2020). Since (±)5(6)-DiHETE inhibited mast cell degranulation, vascular hyperpermeability and itching, it can be a new option to manage AC. Although many antigens are known to cause AC, we evaluated the effect of (±)5(6)-DiHETE in a pollen-induced AC model. However, (±)5(6)-DiHETE has proven beneficial for AC induced by antigens other than pollen, considering its therapeutic action mechanisms.

In this study, we administered (±)5(6)-DiHETE intraperitoneally (300 μg/kg) and topically with eye drops (1 μg per eye). We previously showed that the oral administration of (±)5(6)-DiHETE (150 or 600 μg/kg for 6 days) attenuated DSS-induced colitis in mice. The plasma concentration of (±)5(6)-DiHETE reached 25.05 or 44.79 ng/mL 0.5 h after the administration of 150 or 600 μg/kg, respectively, followed by a gradual decrease. The half-life of (±)5(6)-DiHETE was estimated to be 1.25–1.63 h (Takenouchi et al., 2021). Thus, 25–45 ng/mL of (±)5(6)-DiHETE is thought to be effective for topical application. Although eye drops will be diluted with tears, 1 μg/2 μL of (±)5(6)-DiHETE seemed a reasonable concentration to be effective. For systemic application, results from DSS-induced colitis studies also suggest that 300 μg/kg is a sufficient concentration to be effective. Thus, (±)5(6)-DiHETE presumably attenuated AC by oral administration as well as eye drops.

Supplementation with ω-3 PUFAs has been reported to be beneficial in conjunctivitis. However, a relatively high concentration of ω-3 PUFAs must be ingested over several months, leading to these therapeutic effects. Silva et al. (2018) reported that oral treatment with ω-3 PUFAs containing EPA and DHA (1 capsule of 500 mg/7 kg/day for 6 months) enhanced the effectiveness of topical tacrolimus for the treatment of keratoconjunctivitis sicca in dogs. Hirakata et al. (2019a) reported that a ω-3 diet containing 4% linseed oil (containing 60% linolenic acid) for 2 months alleviated allergic conjunctivitis induced by pollen. In this study, 300 μg/kg (±)5(6)-DiHETE attenuated the pollen-induced AC symptoms. Thus, (±)5(6)-DiHETE may be superior to ω-3 fatty acids in that it can exhibit stronger and more immediate therapeutic effects.

Itching is a major complaint of patients in several allergic diseases. This greatly reduces the QOL of patients. Scratching exacerbates and prolongs allergic diseases by disrupting the epithelial barrier of the conjunctiva, nasal mucosa and the skin. Furthermore, we expect that this anti-itch effect can be applied in diseases other than AC. Most seasonal AC are caused by pollen and approximately 40% of seasonal AC patients develop allergic rhinitis (Yoshisue et al., 2022). Since blueback fish intestines contain (±)5(6)-DiHETE abundantly (Kida et al., 2020), patients could manage allergic rhinitis by ingesting them in some form.

Previously, we found that (±)5(6)-DiHETE inhibited histamine-induced vascular hyperpermeability at least partially by inhibiting the TRPV4 receptor. *In vitro* treatment with (±)5(6)-DiHETE inhibited TRPV4 agonist-induced intracellular Ca^2+^ elevation in TRPV4 over-expressing HEK293T cells (Hamabata et al., 2018b; Kobayashi et al., 2021a).

TRPV4, responsible for sensing osmotic and mechanical signals, is broadly expressed in various tissues; its activation increases intracellular Ca^2+^ levels (Toft-Bertelsen and MacAulay, 2021). Ocular system including conjunctiva (Mergler S et al., 2012; Guarino BD et al., 2020), vascular endothelial cells (Köhler et al., 2006; Earley et al., 2009; Bagher et al., 2012), mast cells (Mascarenhas et al., 2017) and some neurons (Chen et al., 2013) express TRPV4. Some studies have suggested an association between TRPV4 expression and AC symptoms. TRPV4 inhibition reduced C48/80 induced-degranulation of human mast cells (Mascarenhas et al., 2017). Its inhibition also reduces eosinophilia in a toluene diisocyanate (TDI)-induced occupational asthma model (Yao et al., 2019). TRPV4 gene deficiency reduced carbachol-induced tear secretion in mice (Derouiche et al., 2018). It also scratched less after an intradermal injection of pruritogens such as histamine (Chen et al., 2016), serotonin (Akiyama et al., 2016) and C48/80 (Chen et al., 2016). Thus, TRPV4 is involved in various cell activation processes during the onset and progression of AC. It is reasonable to assume that the beneficial effects of (±)5(6)-DiHETE were mediated by the inhibition of TRPV4. Due to the multifactorial causes of AC, a combination of treatment is sometimes used to treat the symptoms of AC (Labib and Chigbu, 2022). (±)5(6)-DiHETE may have possibility to act synergistically with other anti-inflammatory compounds. However, we cannot exclude the possibility of other mechanisms of action of (±)5(6)-DiHETE. Further studies are required to confirm this hypothesis.

In summary, this study demonstrated that the administration of the EPA metabolite (±)5(6)-DiHETE is useful for suppressing the symptoms of allergic conjunctivitis in a mouse model. This lipid is abundant in fish organs (Kida et al., 2020). Therefore, it is necessary to study the administration of (±)5(6)-DiHETE including its extraction and utilization.

## Data Availability

The original contributions presented in the study are included in the article/Supplementary Material, further inquiries can be directed to the corresponding author.
